# Supplementary material for: The Evolution and Characterization of the RNA Interference Pathways in Lophotrochozoa
Source: Genome Biol Evol. 2024 May 7;16(5):evae098. doi: 10.1093/gbe/evae098 (PMC11114477; doi:10.1093/gbe/evae098)
Supplement: evae098_Supplementary_Data [file evae098_supplementary_data.zip › Supplementary_materials.zip!/selected_miRNAs/NoGe_selected_miRNAs/known_miRNA_families_pdf_files/NMRB01003818.1_Notospermus_geniculatus_strain_Ushimado_scaffold3818,_whole_genome_shotgun_sequence_44603.pdf]

Provisional ID	:	NMRB01003818.1_Notospermus_geniculatus_strain_Ushimado_scaffold3818,_whole_genome_shotgun_sequence_44603
Score total	:	106803.8
Score for star read(s)	:	3.9
Score for read counts	:	106795.2
Score for mfe	:	1.7
Score for randfold	:	0
Score for cons. seed	:	3
Total read count	:	209486
Mature read count	:	209327
Loop read count	:	0
Star read count	:	159

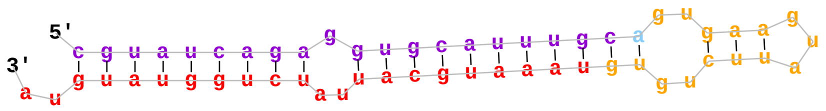

Genome coordinates: NMRB01003818.1 15274-15332

pre-miRNA sequence:
CGUAUCAGAGGUGCAUUUGCCGUAUCAGAGGUGCAUUUGCAGUGAAGUAUUCUGUGUAAAUGCAUUAUCUGGUAUGUA

Mature sequence: UAAAUGCAUUAUCUGGUAUGUA

Aligned mature sequence of the *N. geniculatus* pre-miRNA with the top 5 hits from the BLAST search against the pre-miRNA mirGeneDB set:

```
                              *********************
Nge_Mir-277-P1                uaaaugcauuaucugguaugua-
Esc-Mir-277_3p                uaaaugcauuaucugguaugug-
Npo-Mir-277_3p                uaaaugcauuaucugguaugug-
Obi-Mir-277_3p                uaaaugcauuaucugguauguga
Ovu-Mir-277_3p                uaaaugcauuaucugguauguga
Cte-Mir-277-P1_3p             uaaaugcauuaucugguaugua-
```
